# A survey of parentally reported sleep health disorders in estonian 8–9 year old children

**DOI:** 10.1186/1471-2431-13-200

**Published:** 2013-12-04

**Authors:** Heisl Vaher, Priit Kasenõmm, Veiko Vasar, Marlit Veldi

**Affiliations:** 1 Department of ORL, Tartu University, 1 Kuperjanovi Str., Tartu 51003, Estonia; 2 Tartu University Psychiatry Clinic, 31 Raja Str., Tartu 50417, Estonia

**Keywords:** Sleep disordered breathing, Children, Sleep health, BMI, Parasomnia

## Abstract

**Background:**

Pediatric sleep research is rather new in Estonia. There has not been a comprehensive study of age specific sleep disorders in Estonian children. The aim of this study was to investigate sleep disorders in a sample of Estonian second grade children.

We hypothesized that:

• Children with low BMI are as susceptible to SDB as are children with high BMI.

• Under weight children are susceptible to residual SDB after adenotonsillectomy.

• Parasomnias present with SDB in children.

• Excessive day time sleepiness is a significant symptom which leads parents to suspect sleep disorders in their child.

**Methods:**

A retrospective questionnaire based survey was used to analyze factors influencing sleep, parasomnias, daytime sleepiness, and sleep disordered breathing (SDB). 1065 Pediatric Sleep Questionnaire (PSQ) packets were distributed by post to randomly selected parents of second grade students; 703 (66%) subjects were included in the study group; each parent/guardian participant had one second grade child. Descriptive statistics were used to compare characteristics of SDB symptomatic and healthy children. We used logistic regression to analyze factors influencing sleep and parasomnias in relation to SDB severity. Odds ratios (OR) and 95% CI were used to estimate relative risk.

**Results:**

Parents of children with SDB complaints seem to pay attention to sleep disorders especially when a child is suffering from excessive day time sleepiness. Parasomnias are present simultaneously with SDB and tend to worsen in relation to more severe SDB complaints. Many underweight children have SDB symptoms after adenotonsillectomy.

**Conclusion:**

SDB symptoms are found in both overweight and underweight children. Both groups should be observed, especially in terms of the current focus on overweight children. Careful follow up after SDB treatment is necessary in case of under and overweight children. Parental suspicions regarding SDB are noticeably higher in cases of excessive daytime sleepiness in their children.

## Background

Pediatric sleep disorders are very common phenomena. The etiology, presentation, and symptoms in children can be very different from the symptoms noticed in adults. Sleep problems may affect 10-45% of the pediatric population [1-3]. Studies have shown that sleep problems in children are associated with emotional, behavioral, and cognitive dysfunctions, causing developmental and social difficulties, and other health issues [4,5].

Pediatric sleep disorders not only affect child health, but can impact the well-being of the entire family. Sleep disorders often are under diagnosed because parents, as well as primary health care practitioners, do not notice them or do not consider them dangerous. Unrecognized sleep disorders can lead to academic difficulties, disrupted interpersonal relationships, and impaired behavior and cognition [5,6].

While the importance of healthy sleep to normal pediatric growth and development is widely acknowledged, there are cultural and parental differences regarding how sleep disordered behavior is defined and what are overall accepted pre-sleep activities. Inappropriate pre-sleep activity patterns influence the prevalence of sleep disorders of school-children aged 6–13 years [7]. ICD-10 states that pediatric sleep difficulties may be the result of poor parental discipline in setting sleep times.

Pediatric sleep and research is rather new in Estonia. There has not been a comprehensive study of age specific sleep habits in Estonian children. The aim of this study is to investigate factors influencing sleep, parasomnias, day time sleepiness, and disordered breathing (SDB) symptoms in a sample of Estonian second grade children.

We hypothesized that:

• Children with low BMI are as susceptible to SDB as are children with high BMI.

• Under weight children are susceptible to residual SDB after adenotonsillectomy.

• Parasomnias present with SDB in children.

• Excessive day time sleepiness is a significant symptom which leads parents to suspect sleep disorders in their child.

## Methods

### Study instruments

We used a modified version of “The Pediatric Sleep Questionnaire (PSQ)” in this study [8]. The study period was 2009 September-November. 1065 Pediatric Sleep Questionnaire (PSQ) packets were distributed by post to randomly selected parents of second grade students; 763 (72%) were returned. We were able to use 703 (66%) questionnaires in the study because of incomplete answers in 60 questionnaires. Each parent/guardian filled out the questionnaire regarding one second grade child.

This study was approved by the Human Research Ethics Committee of the University of Tartu; data were coded for privacy. All participant parents/grandparents/guardians had a second grade child in Tartu city/county schools and received a letter which outlined the aims of the study and stated what would be required. Each participating parent/grandparent/guardian provided written consent to participate in the study for themselves and their child.

PSQ was translated from English into Estonian. It was modified to focus on SDB-related complaints. The modified PSQ contains questions about child sleep behavior in the last six months:

– Four open ended questions revealed biometric data: sex, age, height and weight. BMI was calculated according to BMI cut points specified for boys and girls in age groups relevant for this study [9].

– Nine closed questions (answered yes-1/ no-0) reported symptoms of snoring and sleep time breathing disorders:

• snoring;

• loud snoring;

• snoring more than half of sleep time;

• heavy or loud breathing;

• Parental waking because of child’s snoring;

• Parental waking because of child’s cry;

• Disruption of breathing during sleep;

• Moving or shaking the child to continue breathing;

• Removal of the child’s tonsils/adenoids.

– Thirteen closed questions (answered yes-1/no-0):

• parasomnia symptoms (sleep walking episodes; screaming and shouting during sleep; waking up during night because of a bad dream; enuresis during last sixth months; body or head rocking);

• restless sleep symptoms (leg jerking and kicking; moving all around in the bed);

• narcolepsy symptoms (occurrence of sleep without the activity or place of falling asleep; seeing images, hearing sounds while awake; losing control over arms or legs; becoming weak or unsteady when excited, surprised or emotional);

• academic abilities (lacking the ability to concentrate at school; being diagnosed with attention deficit hyperactivity disorder).

– Three questions dealt with sleep times:

• each parent was asked to estimate on a six point scale the typical sleep onset time (20:30 – 1, 21:00 – 2, 21:30 – 3, 22:00 – 4, 22:30 – 5, 23:00–6) and wake-up time (before 6:00 – 5, 6:00 – 4, 6:30 – 3, 7:00 – 4, 7:30 – 5, 8:00 and later – 6) on school nights;

• sleep delay time (>1 h – 4; 45 min - 3; 30 min - 2; 20 min – 1; 0–15 min-0);

• recurrent waking up during the night was estimated on a three point scale (no awakenings - 0; 1–3 awakenings - 1; 4–6 awakenings - 2; more than 6–3).

• To estimate average sleep duration we used the reported time of sleep wake-up minus sleep onset.

– Eight questions dealt with sleep health issues (yes -1/no -0):

• Bedtime resistance (yes -1/no -0);

• Activities in bed other than sleeping (watching TV, reading, listening to music) (yes-1/no-0);

• Regular napping during schooldays (yes -1/no-0);

• Excessive daytime sleepiness (yes -1/no -0);

• Sharing the bedroom (parent - 0/other child -1);

• Regularity of ^“^lights off” timing (yes - 0/no -1);

• Person switching off the lights (parent -0/child-1);

• The number of caffeine drinks consumed (1 or more per day -3; 2–3 per week -2, 1 per week – 1; none – 0).

Two questions dealt with parental views: whether their children had sleep problems (yes -1/ no -0); what was the optimal sleep duration (7 h - 3; 8 h - 2; 9 h – 1; 10 h – 0) for the child were asked.

In this study modest sleep disordered breathing (SDB) complaints were defined as reporting either snoring or heavy breathing; moderate SDB as having snoring and heavy breathing present at the same time; and severe SDB as having breathing pauses together or without snoring, heavy breathing and the need to shake the child to continue breathing. In number of analyses we used SDB categories vs none SDB, whereas for some analyzes all SDB categories where grouped together and compared to no SDB symptoms.

This schema was used categorize and determine the statistical frequency of SDB complaints.

### Data analysis

Statistical analysis was performed using the Statistica 10 software package (Stat.Soft Inc.,Tulsa, OK). The analysis included frequency tables, the chi-square test, and logistic regression. We used logistic regression to analyze sleep related symptoms in relation to SDB severity, adjusted for gender. Odds ratios (OR) and 95% CI were used to estimate relative risk.

## Results

Second grade students, 8–9 years old, were studied in Tartu City and County, Estonia, which has a total population of approximately 151,000.

### Sleep health factors and parasomnias in the Estonian cohort

**Figure 1:**
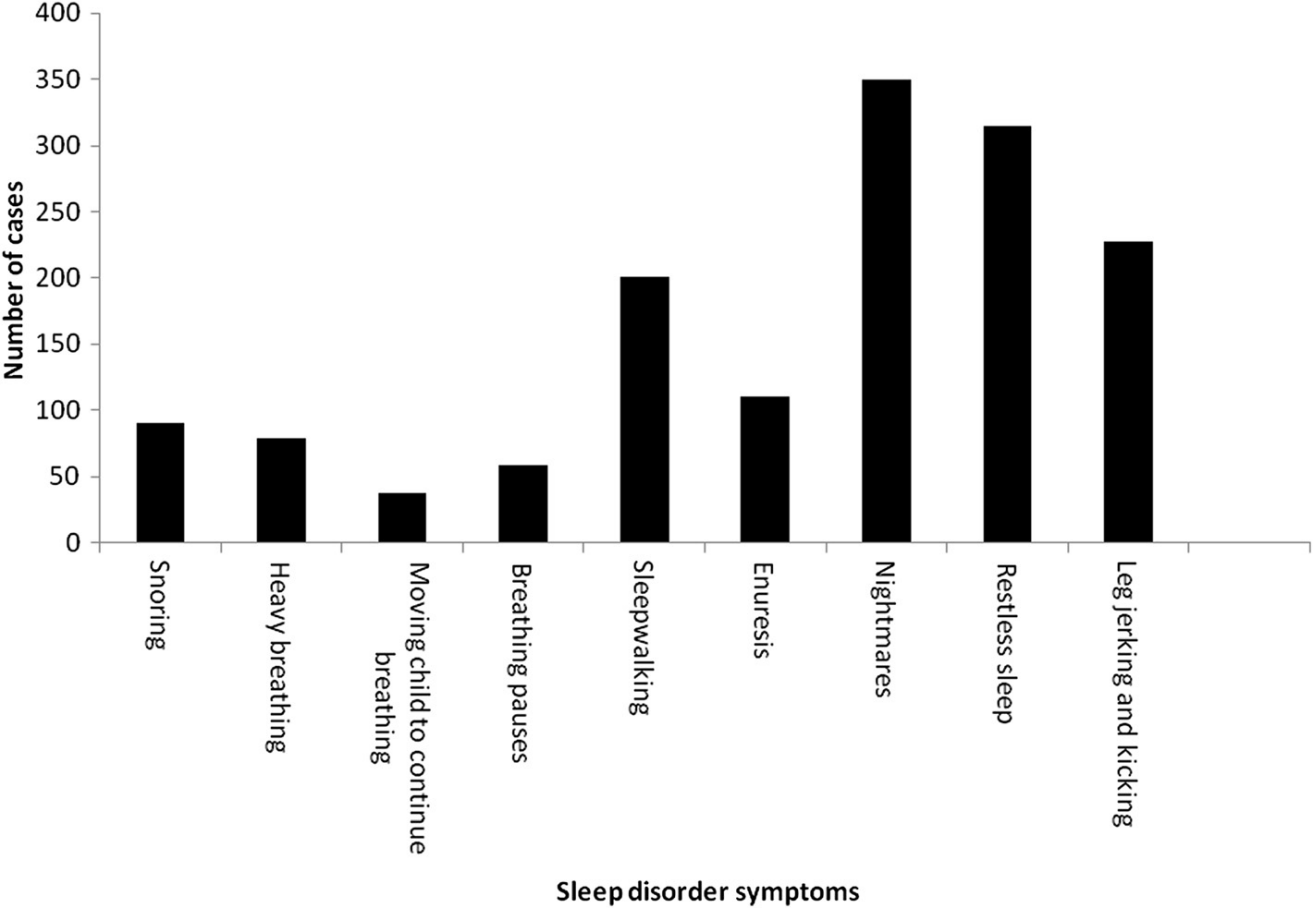
Prevalence of sleep disorder symptoms in overall cohort.

Figure 1 presents the prevalence of sleep disorder symptoms in our study group.

#### *Prevelance of factors influencing sleep health*

The bedroom was shared in 36% (n = 253) of cases. Reluctance at bedtime was reported in 26.3% (n = 185) of subjects. There were 33.5% (n = 236) of subjects with extra activity in bed prior to sleep. Awakenings during night were found in 44% (n = 310) of children. Irregularity of lights off time was reported in 57.5% (n = 405) of cases. We distinguished between SDB and SDB free subjects in Table 1.

**Table 1:** Factors influencing sleep health in SDB and SDB free subjects

**Factors influencing sleep health**	**No SDB (n = 587)**	**SDB (n = 117)**	**p-value (adjusted)**	**OR (95% CI)**
Sharing the bedroom	25% (149)	89% (104)	<.00	22.6 (12.3-41.5)
Reluctance to bedtime	22% (132)	45% (53)	<.00	2.8 (1.9-4.3)
Extra activity in bed prior to sleep	38% (221)	13% (15)	<.00	0.24 (0.14-0.43)
Awakenings during night	42% (247)	54% (63)	<.00	1.6 (1.1-2.5)
Irregular lights off time	52% (309)	82% (96)	<.00	3.9 (2.4-6.5)

*SDB*- sleep disordered breathing.

#### *Parasomnias in the overall cohort*

Table 2 presents parasomnia symptoms in the cohort. Twenty eight percent (n = 201) of children were sleepwalkers. Nightmares were reported in 50% (n = 350) of the study cohort. 32% (n = 227) of subjects had repetitive movements with limbs. Restless sleep was found in 44% (n = 315) of cases. Seven percent (n = 51) of children suffered from enuresis. Parasomnias were reported both in those with SDB symptoms and those SDB free.

**Table 2:** Parasomnias reported in SDB and SDB free cases

**Parasomnia**	**No SDB (n = 587)**	**SDB (n = 117)**	**p-value (adjusted)**	**OR (95% CI)**
Sleepwalking	158 (27%)	43 (37%)	0.04	1.5 (1.0-2.3)
Nightmares	282 (48%)	68 (58%)	0.10	1.4 (0.9-2.1)
Awakenings during night	42% (247)	54% (63)	<.00	1.6 (1.1-2.5)
Repetitive movements with limbs	166 (28%)	61 (52%)	<.00	2.7 (1.8-4.1)
Restless sleep	239 (41%)	76 (65%)	<.00	2.7 (1.8-4.1)
Enuresis	20 (3.4%)	31 (27%)	<.00	9.8 (5.3-18.3)

*SDB*- sleep disordered breathing.

### Sleep disordered breathing (SDB) in the Estonian cohort

16.5% (n = 116) of children had SDB symptoms. Modest SDB was present in 3.7% (n = 26) of boys and 2.3% (n = 16) of girls. Moderate SDB was reported in 1.4% (n = 10) boys and in 0.71% (n = 5) of girls. We found severe SDB in 5.8% (n = 41) of boys and in 2.6% (n = 18) of girls. Gender distribution for those SDB free: 38.4% (n = 270) healthy boys and 54% (n = 317) girls. The overall sex distribution was: 49.4% (n = 347) boys and 50.6% (n = 356) girls. 425 (61%) subjects lived in the county; 278 (39%) resided in the city.

#### *SDB and BMI in our cohort of second graders*

SDB symptoms were analysed in relation to BMI in Table 3. Among underweight children 29.3% (n = 13) of boys and 16.7% (n = 2) girls had severe SDB complaints. 20.7% (n = 17) of overweight and obese boys had severe SDB complaints. Among overweight and obese girls, 11.6% (n = 5) of those reported severe SDB symptoms.

**Table 3:** Severity of SDB in relation to BMI

**Study subjects**	**Modest SDB**	**Moderate SDB**	**Severe SDB**
Normal weight	2% (n = 14)	1.6% (n = 11)	3.1% (n = 22)
Low weight	1% (n = 7)	0.3% (n = 2)	2.1% (n = 15)
Over weight	2.4% (n = 17)	0.1% (n = 1)	2.6% (n = 18)
Obese	0.6% (n = 4)	0.1% (n = 1)	0.6% (n = 4)
	N = 42	N = 15	N = 59

*SDB*- sleep disordered breathing.

#### *SDB symptoms after ENT surgery among the Estonian cohort*

Adenotonsillectomy was performed in 257 cases: adenoidectomy 43% (n = 110); tonsillectomy 33% (n = 85); adenotonsillecomy 23.7% (n = 61). Surgery was performed more frequently in cases with SDB complaints than those without SDB complaints: 45% (n = 53) compared to 35% (n = 204); p = 0.03, OR = 1.6 (1.0-2.3). After surgical treatment, 30% (n = 16/47) of normal weight children had SDB complaints; 46% (n = 12/15) of underweight subjects; and 59.5% (n = 25/54) of overweight children.

#### *SDB severity and sleep health factors*

Parents were asked if their child suffers from any sleep disorder symptoms serious enough to seek medical consultation, giving us a measure of parental perception of sleep health. We then related the parental perception of a sleep problem to the reported sleep health symptoms of their child. Parents reported sleep problems in case of modest SDB 31% of the time (OR = 4.0 95% CI: 2.0 – 8.1); moderate SDB 53.3% (OR = 10.2 95% CI: 3.6 –29.2); severe SDB 81.4% (OR = 39.1 95% CI: 19.2 – 79.2); (p < 0.0001).

#### *SDB severity and factors influencing sleep health*

Sharing the bedroom with another person was statistically significantly related to severe SDB (93.2% OR = 40.4 95% CI: 5.3 – 12.2) and modest SDB (81% OR = 12.5 95% CI: 5.7 – 27.6) in children.

Extra activity in bed prior to sleep appeared to have no relation to SDB complaints.

Reluctance at bed time is statistically significant (p < .0001) in cases of moderate SDB symptoms, 66.7% (OR = 6.9, 95%CI: 2.3-20.5) and in severe cases, 52.5% (OR = 3.8, 95% CI: 2.2-6.6).

The severity of SDB is related to irregularities in lights off time (p < 0.0001): modest 42% (OR = 4.5, 95% CI: 2.0-10.3), moderate 15% (OR = 12.6 95% CI: 1.6-96) and severe 59% (OR = 3.5, 95% CI: 1.8 -6.8) SDB complaints.

Excessive daytime sleepiness was statistically significant in relation to SDB complaints (p < .0001): moderate SDB, 86.7% (OR = 10.5, 95% CI: 4.2 - 26.7); severe SDB, 91.5% (OR = 8.1, 95% CI: 2.7 - 24.4).

Difficulty in school concentration was statistically related to SDB severity (p < .0001): modest SDB, 81% (OR = 21.5, 95% CI: 9.6 - 47.8); moderate SDB, 80% (OR = 20.2, 95% CI: 5.6 - 72.9); severe SDB, 88.1% (OR = 37.5, 95% CI: 16.6 - 85.1).

Parental reporting of their child’s sleep problems increases with the co-presentation of SDB 81.3% (p < 0.0001, OR = 6.3 95% CI: 2.5 – 15.9), and excessive daytime sleepiness 42.9% (p = 0.0012, OR = 10.1 95% CI:2.8 – 36.0).

#### *SDB severity and parasomnias*

The prevalence of parasomnias increased in moderate and severe SDB cases (Table 4).

**Table 4:** Parasomnias related to SDB severity

**Parasomnias**		**SDB groups**				
	**No SDB (n = 587)**	**Modest (n = 42)**	**Moderate (n = 15)**	**Severe (n = 59)**	**p**	**OR (95%CI)**
Sleepwalking	27% (158)	26% (11)	47% (7)	42% (25)	0.03	2.0 (1.2-3.5)
Nightmares	48% (282)	50% (32)	87% (13)	58% (34)	0.04	7.0 (1.6-31)
Repetitive movements with limbs	28% (166)	29% (12)	73% (11)	64% (38)	<.00	4.6 (2.6-8.1)
Restless sleep	41% (239)	45% (19)	80% (12)	76% (45)	<.00	4.7 (2.5-8.7)
Enuresis	15% (85)	19% (8)	60% (9)	14% (8)	0.00	8.9 (3.1-25)

*SDB*- sleep disordered breathing.

## Discussion

### Children with low BMI are as susceptible to SDB as are children with high BMI

In the present study, we found that parents of Estonian 8–9 year old children reported the rates of sleep disorders and SDB complaints in the mean range of what has been reported in the literature. Snoring has been found in 3-12% of children and breathing pauses referring to sleep apnea in the range of 1.2-5.7% [8,10,11]. Our findings of SDB complaints were somewhat higher. This could be explained by the subjective source of the data. We did notice a gender difference in reporting SDB complaints as also found by other authors [10,12], showing that boys are somewhat more prone to serious SDB complaints.

Obesity, underweight status and tonsillar hypertrophy have been found to be risk factors for SDB in several studies [13-17]. Obesity is defined as BMI > 95th percentile for age and gender [9,18]. In our study overweight children composed up to one third of those with SDB complaints. We also noticed a remarkable number of underweight subjects with severe SDB complaints.

### Under weight children are susceptible to residual SDB after adenotonsillecomy

We found that almost a half of children with SDB had ENT surgery; however, SDB symptoms remained. Residual SDB has been reported in the range of 13-73%. For overweight children, the surgical treatment of SDB carries a risk of residual SDB [19]. We did not find studies focusing on underweight children with residual obstructive sleep apnea after adenotonsillectomy. There appears a need to follow up with underweight subjects for residual SDB after adenotonsillectomy.

### Parasomnias present with SDB in children

Parasomnias are frequent complaints among pediatric populations, being undesirable events that accompany sleep in conjunction with other sleep disorders such as SDB [20]. It is common for several parasomnias to occur in one patient: rhythmic movements, quick jerks, sleep talking, sleepwalking, sleep terrors, nightmares and excessive daytime tiredness have been found to coincide with SDB [21]. In our study sleepwalking was the more frequent finding in SDB subjects. Mindell et al. 2003 reports the incidence of pediatric sleep walking in the range of 15-40% of the population. In our cohort, the prevalence of parasomnias is higher among those with moderate and severe SDB. Repetitive movement of the limbs and reports of restless sleep was especially common.

### Excessive day time sleepiness is a major symptom which leads parents to suspect sleep disorders in their child

Several studies deal with factors influencing sleep and their consequences for pediatric sleep [7]. We focused on sleep health related variables often influenced by parental behavior regarding the child’s sleep needs, such as extra activity in bed prior to sleep, reluctance at bed time, sharing the bedroom and irregularities of switching the lights off. Sharing the bedroom was prevalent in about one quarter of study subjects; in cases of SDB symptoms, bedroom sharing tends to be high. This could be explained by socioeconomic status and cultural traditions [22]. We suggest that parents sleeping in the same room as their child may notice SDB symptoms more frequently.

Excessive daytime sleepiness and sharing the bedroom can be associated with an increased in the severity of SDB complaints based on the data from our study. Excessive day time sleepiness is also a frequent complaint in non SDB children [23]. The prevalence of daytime sleepiness has been reported between 4.9-10% among 5–18 year olds [3,24-26]. Almost half of our study cohort reported excessive day time sleepiness either sometimes or always. SDB complaints increased the likelihood of being excessively tired during the day. Focusing issues at school did not gain that much attention from parents compared to daytime somnolence when child also had SDB complaints.

Extra activities in bed prior to sleep were not a frequent complaint regarding children with SDB symptoms. Spruyt et al. 2005 found that every fourth child goes to bed reluctantly. Our findings were the same in case of children without SDB complaints, however, reluctance to bed time and struggling with regular lights off time are more prevalent in cases of SDB complaints. Irregular lights off time also increased the frequency of awakenings during the night. We found noncompliant behavior (termed bedtime resistance in this study) in approximately a quarter of healthy subjects and extra activities prior to sleep in more than half of this group. However, subjects with SDB complaints had greater bedtime resistance than the healthy group. The SDB group had fewer extra activities in bed prior to sleep. In the literature, there is a wide variation in the frequency range of bed time resistance: 3.6 to 52.6% [3,7,24,26].

Parents feel that pediatric sleep disorders are not a major health concern and are a self-resolving problem [24,27]. In one study it was noted that despite sleep disorders as a frequent complaint, only 6.7% of parents specifically reported that their children had sleep difficulties [28]. We did notice in our cohort that SDB complaints are alerting to parents; in case of serious complaints parents do feel there is a sleep problem. But this did not apply for other sleep disorders like parasomnias alone without SDB.

### Study limitations

Limitations of current study should be noted. A proportion of subjects were excluded from analyses because of incomplete questionnaires. Parents/guardians reported their observations of their children’s sleep related behavior. The collection of socioeconomic data was not part of the study design.

## Conclusion

In our cohort of Estonian children, SDB frequency is in the same range of what has been reported by other authors. Our data suggest that attention regarding pediatric SDB symptoms should be paid to underweight children as well as those that are overweight. Parasomnias are present simultaneously with SDB and tend to worsen in relation to more severe SDB complaints. Parents of children with SDB complaints seem to pay attention to sleep disorders especially when a child is suffering from excessive day time somnolence. However, parental awareness regarding pediatric sleep health symptoms needs improving.

## Competing interest

Heisl Vaher, Priit Kasenõmm, Veiko Vasar and Marlit Veldi have no financial support from or author involvement with an organization(s) with financial interest in the subject matter of the paper. The authors declare that they have no competing interests.

## Authors’ contributions

HV, MV, VV conceived and designed the study. HV and MV collected the data. HV and PK analyzed the data. HV, MV and PK drafted the manuscript. All authors revised and approved the final manuscript.

## Pre-publication history

The pre-publication history for this paper can be accessed here:

http://www.biomedcentral.com/1471-2431/13/200/prepub

## Support

This research was supported by the Estonian Science Foundation (SF0180125s).
